# The predictive value of resting heart rate following osmotherapy in brain injury: back to basics

**DOI:** 10.1186/2008-2231-20-102

**Published:** 2012-12-30

**Authors:** Mahsa Hasanpour Mir, Fardin Yousefshahi, Mohammad Abdollahi, Arezoo Ahmadi, Atabak Nadjafi, Mojtaba Mojtahedzadeh

**Affiliations:** 1 Department of Pharmacotherapy, Faculty of Pharmacy, Tehran University of Medical Sciences, Tehran, Iran; 2 Department of Anesthesia, Faculty of Medicine, Tehran University of Medical Sciences, Tehran, Iran; 3 Department of Toxicology and Pharmacology, Faculty of Pharmacy and Pharmaceutical Sciences Research Center, Tehran University of Medical Sciences, Tehran, Iran; 4 Anesthesiology & Intensive Care Department, School of Medicine, Tehran University of Medical Science and Health Services, Tehran, Iran

**Keywords:** Heart rate, APACHE II score, SOFA score, GCS score, Head injury

## Abstract

**Background:**

The importance of resting heart rate as a prognostic factor was described in several studies. An elevated heart rate is an independent risk factor for adverse cardiovascular events and total mortality in patients with coronary artery disease, chronic heart failure, and the general population. Also heart rate is elevated in the Multi Organ Dysfunction Syndrome (MODS) and the mortality due to MODS is highly correlated with inadequate sinus tachycardia.

To evaluate the value of resting heart rate in predicting mortality in patients with traumatic brain injury along scoring systems like Acute Physiology and Chronic Health Evaluation(APACHE II), Sequential Organ Failure Assessment (SOFA) and Glasgow Coma Score (GCS).

**Method:**

By analyzing data which was collected from an open labeled randomized clinical trial that compared the different means of osmotherapy (mannitol vs bolus or infusion hypertonic saline), heart rate, GCS, APACHE II and SOFA score were measured at baseline and daily for 7 days up to 60 days and the relationship between elevated heart rate and mortality during the first 7 days and 60th day were assessed.

**Results:**

After adjustments for confounding factors, although there was no difference in mean heart rate between either groups of alive and expired patients, however, we have found a relative correlation between 60^th^ day mortality rate and resting heart rate (P=0.07).

**Conclusion:**

Heart rate can be a prognostic factor for estimating mortality rate in brain injury patients along with APACHE II and SOFA scores in patients with brain injury.

## Introduction

In critical care units, patients with moderate to severe brain injury are often intubated and sedated to diminish the workload of the brain. Agitation or restlessness is common in these patients and can be associated with fever, tachycardia, hypertension, and diaphoresis. This exaggerated stress response, known as sympathetic storming, occurs in 15% to 33% of patients with severe traumatic brain injury who are comatose and Glasgow coma scale is less than 8. Sympathetic storming can occur within the first 24 hours after injury or up to weeks later [1]. The precise mechanism for the increase in activity of the sympathetic nervous system is unknown, but the increased activity is thought to be a stage of recovery from severe traumatic brain injury [2].

The importance of resting heart rate as a prognostic factor was described in a retrospective analysis in 1945 [3]. A high heart rate is an independent risk factor for adverse cardiovascular events and total mortality in patients with coronary artery disease [4], chronic heart failure [5], and the general population [6]. Reduction in heart rate is associated with an improvement in prognosis in patients with cardiovascular disease [7].

Acute Physiology and Chronic Health Evaluation (APACHE II) evaluates cost/benefit of intensive care and performance of ICU work [8,9] and categorizes patients or groups of patients according to the severity of their diseases and also predicts the outcome and response to therapy and compares patient groups in acutely ill patients [10].

The sequential organ failure assessment (SOFA) score is a scoring system to determine the extent of a person's organ function or the rate of failure in critically ill patients and to compare patients that would benefit clinical interventions and differentiate the surgical patients who require either shorter (less than 24 hours) or longer ICU admission time [11].

The aim of this study is to evaluate the predictive value of heart rate in traumatic brain injury patients following osmotherapy.

## Methods

This study was an open label randomized clinical trial (clinical registration ID: 201011055107N1) and conducted at Sina trauma center in Tehran (Iran) between October 2008 and May 2011. The study had received ethical approval from the ethic committee of Tehran University of Medical Sciences and health services (TUMS). Of 33 patients, Group A (n=10) received mannitol 20% as a bolus of 1 g/kg, Repeated dosing was given at 0.25 to 0.5 g/kg as needed, generally every six hours, Group B (n=11) received 125 cc HTS (Hyper Tonic Saline) 5% as bolus in 1hour every 6 hour and Group C (n=12) received 500 cc HTS 5% as infusion during 24 hours.

For each patient a set of variables included initial GCS, APACHE II, SOFA scores and heart rate were collected and assessed at baseline and daily during the course of ICU stay.

Length of ICU and hospital stay, mortality at 7^th^ and 60^th^ days were recorded for all patients for outcome evaluation. Blood samples were drawn on admission and first, second and third day.

### Statistical analysis

Qualitative variables were recorded by frequency and percent and compared by Fisher's exact test. Quantitative variables were recorded by Mean ± SD (Standard Deviation). ANOVA and Kruskal-wallis test were used, for comparing quantitative variables in three groups, when was appropriate. For Survival analysis, Kaplan-Meier method was applied and for comparison between groups in survival, Log-Rank test was selected. T-test was conducted to compare mean heart rate, mean APACHE II, mean SOFA and mean GCS after 7 and 60 days between alive and deceased groups. Regarding variables like baseline APACHE II, SOFA, GCS scores and baseline heart rate, Logistic regression was performed to find correlation between heart rate and 60 days mortality. P value of less than 0.05 was considered significant.

## Results

We assessed 39 consecutive patients with moderate and severe TBI (Traumatic Brain Injury), 6 of them were ineligible. From 33 remaining patients, 10 of them received mannitol (group A), 11 patients received HTS as a bolus (group B) and 12 patients as a continuous infusion of HTS (group C).

APACHE II, SOFA and GCS scores at baseline and also during ICU and hospital stay are shown in Table 1. Evaluation of 60 days survival between patients (Table 2) have been demonstrated that there was no significant difference in 60 days survival of patients for different treatment groups (P=0.1).

**Table 1:** Acute Physiology and Chronic Health Evaluation, Glasgow Coma Scale and Sequential Organ Failure Assessment data at baseline

	**Mannitol**	**Bolus of HTS**	**Infusion of HTS**	**P value**
Initial GCS	6.10±3.07	8.00±2.23	1.83±6.91	0.06^a^
Initial SOFA	1.94±6.30	6.27±1.67	7.08±2.53	0.52^a^
Initial APACHE II	18.70±5.29	18.18±3.48	19.08±5.14	0.68^a^
LoICU stay(day)	16.9±9	14.2±12	11.17±7.7	0.5^a^
LoH stay(day)	20.7±21.25	18.18±12.4	18.09±1.84	0.9^a^

a: One way ANOVA.

* P<0.05 considered significant.

*HTS*: hypertonic saline, *GCS*: Glasgow Coma Scale, *SOFA*: Sequential Organ Failure Assessment, *APACHE II*: Acute Physiologic and Chronic Health Evaluation, *LoICU*: Length Of Intensive Care Unit, *LOH*: Length Of Hospital.

**Table 2:** 60 days survival of Patients in three groups

**Group**	**Survival**	**Mean of survival**	**Std. Error**	**95% Confidence Interval**	**P value**
**Mannitol**	21%	28.9	8.5	12.06-45.7	**0.1**
**Bolus of HTS**	82%	40.2	4.9	30.5-49.9	
**Infusion of HTS**	65.5%	46.8	9.2	28.7-64.8	
**Overall**	48%	41.9	5.9	30.4-53.5	

Kaplan-Meier method for Survival analysis and Log-Rank test for comparison between groups.

HTS: Hyper Tonic Saline.

*P<0.05 considered significant.

There was a significant correlation between mean APACHE II, SOFA and GCS scores in treatment groups (P=0).

There was no significant difference in mean heart rate, mean APACHE II and SOFA scores between deceased and alive patients after 7 days (Table 3), but a significant difference was shown in mean GCS score between these two groups (P=0.04).

**Table 3:** **Correlation between heart rate, acute physiology and chronic health evaluation, neurologic and organ function severity scores and 7**^**th**^**day mortality in dead and alive groups**

**Heart rate**	**Total**	**Live**	**Dead**	**P value Between alive and dead patients**
Baseline	93.27±17.17	91.35±17.04	104±15.16	0.13
The 1^st^ day	94±18.42	93.64±18.78	96±18.16	0.79
The 2^nd^ day	90.21±17.01	91.14±17	85±18.02	0.46
The 3^th^ day	88.27±15.75	86.89±14.99	96±19.49	0.24
The 4^th^ day	89.35±18.23	87.85±17.27	103.33±25.16	0.16
The 5^th^ day	92.41±18.29	91.66±18.12	102.50±24.74	0.42
The 6^th^ day	89.07±16.92	89.03±17.24	90	0.95
The 7^th^ day	87.82±13.14	87.74±13.38	90	0.87
**APACHE II**				
Baseline	17.68±4.58	17.44±4	19±7.48	0.49
The 7^th^ day	15.22±4.90	15.26±5	14	0.80
**SOFA**				
Baseline	6.09±2.11	5.92±1.96	7±2.91	0.30
The 7^th^ day	5.11±3.14	5.15±3.19	4	0.72
**GCS**				
Baseline	7.43±2.75	7.69±2.76	5.75±2.21	0.19
The 7^th^ day	7.96±3.45	8.50±3.63	5.50±0.70	0.30
**Mean heart rate**	90.79±14.22	89.90±14.27	95.76±14.34	0.40
**Mean APACHE II**	16.22±4.61	15.70±3.62	19.08±8.36	0.13
**Mean SOFA**	5.90±2.31	5.63±2.08	7.44±3.19	0.10
**Mean GCS**	7.63±2.79	8.03±2.73	5.36±2.07	0.04

*APACHE II*: Acute Physiologic and Chronic Health Evaluation, *SOFA*: Sequential Organ Failure Assessment, *GCS*: Glasgow Coma Scale.

*P<0.05 considered significant.

**Figure 1:**
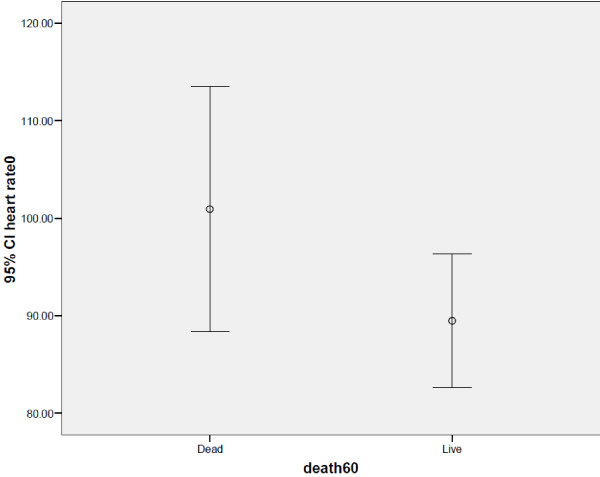
**Correlation between elevated baseline heart rate and 60th day mortality.** This figure shows that baseline heart rate is different between these two groups (P=0.07) and is higher in alive group.

There was a significant difference between expired and alive patients in mean APACHE II (P=0.005), SOFA (P=0.006) and GCS scores (P=0.000) after 60 days. Also baseline heart rate was different between these two groups (P=0.07) as presented in Table 4 and Figure 1.

**Table 4:** Correlation between heart rate, acute physiology and chronic health evaluation, neurologic and organ function severity scores and 60th day mortality in dead and alive groups

**Heart rate**	**Total**	**Live**	**Dead**	**P value Between alive and dead patients**
Baseline	93.27±17.17	89.45±15.41	100.90±18.68	0.07
The 1^st^ day	94±18.42	90.54±16.88	100.90±20.22	0.13
The 2^nd^ day	90.21±17.01	89.86±13.30	90.90±23.53	0.89
The 3^th^ day	88.27±15.75	84.13±9.31	96.54±22.34	0.10
The 4^th^ day	89.35±18.23	86.13±15.17	97.22±23.33	0.12
The 5^th^ day	92.41±18.29	87.38±9.13	105.62±28.71	0.11
The 6^th^ day	89.07±16.92	85.04±9.15	101.14±27.97	0.18
The 7^th^ day	87.82±13.14	85.19±9.24	95.71±19.88	0.20
**APACHE II**				
Baseline	17.68±4.58	16.61±3.51	19.72±5.78	0.06
The 7^th^ day	15.22±4.90	13.80±3.94	19.28±5.40	0.008
**SOFA**				
Baseline	6.09±2.11	5.18±1.33	7.90±2.25	0.002
The 7^th^ day	5.11±3.14	4.15±1.69	7.85±4.67	0.08
**GCS**				
Baseline	7.43±2.75	7.90±2.68	6.33±2.73	0.15
The 7^th^ day	7.96±3.45	8.90±3.66	5.62±0.91	0.001
**Mean heart rate**	90.79±14.22	87.26±9.41	97.84±19.46	0.11
**Mean APACHE II**	16.22±4.61	14.68±2.96	19.30±5.84	0.005
**Mean SOFA**	5.90±2.31	4.93±1.23	7.85±2.77	0.006
**Mean GCS**	7.63±2.79	8.61±2.80	5.66±1.45	0.000

*APACHE II*: Acute Physiologic and Chronic Health Evaluation, *SOFA*: Sequential Organ Failure Assessment,

GCS: Glasgow Coma Scale.

*P<0.05 considered significant.

Regarding to the performed Logistic regression (baseline scores and three groups of osmotherapy were assessed) there was a correlation between baseline heart rate and 60^th^ day mortality (P=0.07). In the other words when the Heart Rate were elevated (Table 5), 60 days mortality was also increased significantly (OR=0.91). Three means of osmotherapy did not affect 60^th^ day mortality. We have also found that baseline SOFA and GCS scores had significant correlation with 60th day mortality (P=0.01 and 0.15 respectively). As a result, by increasing of SOFA score, 60^th^ day mortality increased moderately (OR=0.33) and by decreasing of GCS score, 60^th^ day mortality increased significantly (OR=1.93). On the other hand, heart rate and APACHE II score of 7^th^ day had correlation with 60^th^ day mortality (P=0.07 and 0.04 respectively) meaning that by increasing of 7^th^ day heart rate, 60^th^ day mortality increased significantly (OR=0.88) and when APACHE II score of 7^th^ day increased, 60^th^ day mortality increased moderately (OR=0.61).

**Table 5:** Logistic regression between 60th day mortality and parameters affecting it in treatment groups

**Mortality (60 days)**	**Variable**	**P value**	**OR**
	**Heart rate (Baseline)**	0.07	0.91
	**SOFA (Baseline)**	0.01	0.33
	**GCS (Baseline)**	0.15	1.93
	**Heart rate (the 7**^**th**^**day)**	0.07	0.88
	**APACHE II (the 7**^**th**^**day)**	0.04	0.61

*APACHE II*: Acute Physiologic and Chronic Health Evaluation, *SOFA*: Sequential Organ Failure Assessment, *GCS*: Glasgow Coma Scale.

*P<0.05 considered significant.

## Discussion

Recent studies show that evaluation of APACHE II and TNF-α in the first day and APACHE II and IL-6 in the third and seventh days of severe septic patients are independent outcome predictors and suggest that IL-6 and APACHE II are useful cytokine and scoring systems respectively in prediction of mortality and clinical evaluation of these patients [12].

Although reducing heart rate is a therapeutic target to improve outcomes in cardiovascular patients [13], the question of transferability to critically ill traumatic brain injury patients remains unanswered. Nevertheless as a result of elevated intracranial pressure, heart rate might be even lowered than the therapeutic range for instance in spinal cord injury and neurogenic shock, heart rate could be less than 60 bpm. The same thing is true for sever traumatic brain injury where the Glasgow Coma Score is less than 8 and cerebral perfusion pressure is below 60 mmHg and intracranial pressure is beyond 20 mmHg. The results of our study have indicated that there is a correlation between baseline heart rate and 60^th^ day mortality but mean heart rate was not different between groups of alive and diseased patients. To our knowledge, this was the first study which was evaluated the role of a physiological parameter (heart rate) as a predicting tool for estimating mortality.

There are number of studies which have evaluated the reliability of different scoring systems in predicting morbidity and mortality in ill patients. Chen et al. had shown that there was a good correlation between expected mortality predicted by the APACHE II scoring system and observed mortality and APACHE II was useful for evaluating ICU performance and risk stratification [14]. Rehman has demonstrated that this score did not show any correlation between predicted mortality and observed mortality in critically ill patients but it was useful for substantial reduction in suffering and cost [15]. In our study, use of APACHE II score was useful for predicting prognosis and there was a significant difference in mean APACHE II score between groups of deceased and alive patients. Therefore, this scoring system not only allows a grading of disease severity but also dependably describe patient prognosis.

Vincent’s study had shown that the SOFA score was a simple, but effective method to describe organ dysfunction/failure in critically ill patients and regular, repeated scoring enables patient condition and disease development to be monitored and better understood. Also, it may enable comparison between patients that would benefit clinical trials [11]. In our study, this score was useful for evaluating disease development because of significantly difference in mean SOFA score between diseased and alive patients.

Ferreira’s study had shown that both mean and highest SOFA scores were good indicators of prognosis and have predicted outcome usefully during the first few days of ICU admission. Independent of the initial score, an increase in SOFA during the first 48 hours in the ICU has predicted a mortality rate of at least 50% [16]. In our study a significant correlation between baseline SOFA score and 60 days mortality was observed.

Adverse impact of increased heart rate has different pathophysiological explanations such as increased myocardial oxygen demand and reduced coronary blood flow due to shortened diastole. Also, it has been shown that there is an increased disposition to rupture atherosclerotic plaques at elevated heart rates [17].

Endotoxin, which is a frequent cause of sepsis and consecutive MODS and increased in heart failure, directly affects the hyperpolarization-activated cyclic nucleotide gated (HCN) channels mediating the pacemaker of human cardiomyocyte [18]. Apart from that, endotoxin sensitizes the HCN (Hyperpolarization-activated Cyclic Nucleotide) channels for sympathetic stimulation and increasing heart rate. Therefore, endotoxin increases heart rate and reduces heart rate variability in chronically instrumented mice and blocking sympathetic and vagal activity induces bradycardia. Totally endotoxin induces inadequately high heart rate and narrowed heart rate variability which causing cardiac and autonomic dysfunction and indicating poor prognosis in patients with MODS [13].

Elevation of heart rate leads to increasing of intracranial pressure. Increased intracranial pressure reflects the presence of mass effect in the brain and is associated with a poor outcome with acute neurological injury. If sustained, it has a negative effect on cerebral blood flow and cerebral perfusion pressure, can cause direct compression of vital cerebral structures, and can lead to herniation. The management of the patient with increased intracranial pressure involves the maintenance of an adequate cerebral perfusion pressure, prevention of intracranial hypertension, and optimization of oxygen delivery [19].

If the metabolic demands exceed the supply, cerebral ischemia will ensue, leading to irreversible neurological damage [20].

In addition, elevated ICP may intensify the processes involved in secondary brain injury, negatively affecting outcomes. It is well established that intracranial hypertension negatively affects morbidity and mortality [20].

Many complications are commonly associated with TBI, such as deep vein thrombosis (DVT), hyperglycemia, and excessive protein loss. By promoting optimal ICP (Intra Cranial Pressure) and CPP (Cerebral Perfusion Pressure) DVT (Deep Venous Thrombosis) complications, inadequate nutrition, detrimental hyperglycemia, and seizures in adults with severe TBI can be prevented [21].

In the multifactor primary prevention trial in GÖteborg, a clear correlation between resting heart rate and all-cause-mortality was shown. Compared with individuals with a heart rate <60 bpm, a heart rate >90 bpm in participants was associated with a two- to three-fold elevated mortality [22]. Another trial which followed up patients with coronary heart disease has shown a significant correlation of elevated heart rate with mortality [4]. In GISSI-2 trial, the heart rate of patients with acute myocardial infarction at discharge without atrial fibrillation proved to be an independent predictor of survival and 6-month mortality was higher for heart rate>100 bpm in compared to heart rate<60 bpm [23]. In addition to heart rate at baseline, the mortality rate also depends on the extent of heart rate reduction by using beta-blocker [7].

In our study, a correlation between elevated baseline heart rate and 60^th^ day mortality was shown; But this was not true about 7^th^ day mortality. Mortality in brain injury is mainly dependent to severity of the injury and elevated baseline heart rate is also a reflection of disease severity.

The relative correlation between baseline Heart Rate and 60^th^ day mortality is not observed in last 7 days. These differences may be a reflection of modifications by medications or other therapeutic measurements during acute phase of treatment.

A fast heart rate on the day of admission was an independent and early predictor of death due to MODS in a prospective, multicenter, observational cohort trial of high-risk patients with noncardiac surgery [24].

Following cardiac arrest, therapeutic hypothermia by decreasing heart rate and cathecolamine storm may also improve neurological recovery [25,26].

Based on the results of the BEAUTIFUL-trial, the patients with stable coronary heart disease and heart failure with a heart rate >70 bpm have a worse prognosis. Above a heart rate of 65 bpm, every increase in heart rate of 5 bpm was associated with a higher risk of all-cause-mortality, hospitalization because of worsening heart failure, hospitalization because of myocardial infarction and hospitalization because of coronary revascularization [27].

Increasing data indicate treatment with beta blockers might improve survival after traumatic brain injury. The optimal heart rate range for these patients is unknown. Admission heart rate in moderate to severe TBI patients was analyzed to determine if a specific range is associated with decreased mortality. After isolated moderate to severe TBI, HR <50, 50–59, 60–69, and > or =110 were independent predictors of increased mortality compared with HR 80–89. HR outside the range 70–109 could serve as a marker for aggressive resuscitation. As mortality increased significantly with HR <50, 50–59, and 60–69, avoiding HR <70 in patients with moderate to severe TBI recommended [28].

## Conclusion

Although, ICU scoring systems like APACHE II and SOFA are useful tools that are used to quantify severity, estimate mortality, and allow comparisons among patients, the experiences of authors in ICU in critically ill patients bring up some limitations of such score in prediction of outcome of individual patients [29-32] a notion remaining to be proved in future by use of a major systematic review and meta-analysis technique. Resting heart rate is an independent predictor of sudden cardiac death and there is a correlation between heart rate and mortality in critically ill patients. Baseline heart rate, as a physiological parameter can predict mortality rate along with scores in patients with brain injury.

## Competing interest

This paper is the outcome of the first author thesis study and was supported by TUMS. Since, the third author is the Editor-in-Chief of the journal; all review process and decisions on the submission were managed by one of Section Editors.

## Authors’ contributions

MH was responsible for patient's selection, data gathering and analysis. FY was responsible for patients' monitoring and data collections. MA was one of supervisors and helped in completing the idea, design of study, and editing the manuscript. AA and AN helped on data gathering and patients selections. MM was the main supervisor. All authors read and approved the final manuscript.
